# Congenital Exophthalmic Goitre

**Published:** 1890-07

**Authors:** A. H. Tufts


Congenital Exopthalmic Goitre.—March 29th, Mrs. R——, age forty, was delivered of a male child, weighing about nine pounds. Position left occipito posterior, presentation vertex and right hand, cord once around the neck. Labor in other respects normal. The mother had previously borne six healthy, living children. She now has a large exopthalmic goitre. The child was likewise possessed of one which was larger, according to the size of the child, than the mother's. The child gasped but a few times after delivery, and the usual methods failed to establish respiration.—A. H. Tufts in *Northwestern Lancet.*

